# Endoscopic peroneus brevis tendon transfer for chronic ruptures of the Achilles tendon: surgical technique

**DOI:** 10.1186/s13018-024-04534-0

**Published:** 2024-02-10

**Authors:** Nicola Poeta, Nicola Maffulli, Fabrizio Bucolo, Christel Charpail, Filippo Migliorini, Stéphane Guillo

**Affiliations:** 1 Department of Medicine, Surgery and Dentistry, University of Salerno, Baronissi, SA Italy; 2 Department of Medicine and Psychology, University Hospital Sant’Andrea, University La Sapienza, 00185 Rome, Italy; 3 Faculty of Medicine, School of Pharmacy and Bioengineering, Keele University, Stoke-on-Trent, ST4 7QB England; 4 Centre for Sports and Exercise Medicine, Barts and the London School of Medicine and Dentistry, Queen Mary University of London, Mile End Hospital, 275 Bancroft Road, London, E1 4DG England; 5 SOS Pied Cheville Bordeaux-Mérignac-Bruges, Clinique du Sport, 4 rue Georges Negrevergne, 33700 Mérignac, France; 6 Department of Orthopaedic, Trauma, and Reconstructive Surgery, RWTH University Medical Centre, Pauwelsstraße 30, 52074 Aachen, Germany; 7 Department of Orthopedics and Trauma Surgery, Academic Hospital of Bolzano (SABES-ASDAA), Teaching Hospital of Paracelsus Medical University, 39100 Bolzano, Italy

**Keywords:** Chronic Achilles tendon rupture, Peroneus brevis transfer, Endoscopic surgery

## Abstract

Chronic Achilles tendon rupture is usually defined as a rupture diagnosed 4–6 weeks after injury. The management of chronic Achilles tendon rupture (CATR) is a topic of hot debate, and no consensus has been achieved. Surgical management of CATR is recommended. Several approaches, techniques, and grafts have been described. Open techniques carry a high risk of wound breakdown, infection, and necessitate long rehabilitation times. Surgical techniques with smaller incisions to reduce the risk of scar fibrosis, pain, and infection are becoming common. The ipsilateral tendon of the hallux flexor longus and the peroneus brevis is commonly used. Endoscopic transfer of the peroneus brevis tendon is an innovative alternative to other procedures, with comparable results of other autografts even in elite athletes. The tendon of the peroneus brevis is harvested by tendoscopy before performing a calcaneal tendon endoscopy and fixing the graft in a calcaneal tunnel using an interference screw. After surgery, an anterior splint is placed for 3 weeks with immediate forefoot weight bearing. The rehabilitation starts on the 15th postoperative day.

## Introduction

In chronic Achilles tendon ruptures (CATR), conservative management leads to poor results because of loss of continuity between the gastrosoleus complex and the calcaneus [1–4]. Most authors recommend surgery [5]. Many reconstruction procedures have been described: tendon grafts, turndown fascia flaps, transfer of local tendons, and synthetic materials [6–8]. However, there is no evidence of the superiority of one procedure over the others [9].

Minimally invasive surgical techniques result in lower rates of wound breakdown, infections, and complications compared to open techniques [10, 11]. Less extensive soft tissue exposure and small incisions improve recovery and shorten rehabilitation times [11–13]. Plantar flexion strength deficits and decreased calf muscle bulk may persist following surgery but are not clinically relevant [3]. Peroneus brevis tendon transfer for CATR was popularized in 1974 [14]. Since then, it has been used successfully in patients with large defects of the Achilles tendon, and minimally invasive procedures have been described [12, 15]. Here, we report a technique for the endoscopic transfer of the peroneus brevis tendon to the calcaneus for the management of CATR. We hypothesized that this technique, given its reduced exposure, is associated with a lower risk of infection.

## Surgical procedure

This intervention is recommended in patients with CATR. Contraindications include ankle instability or peroneal tendon lesions, especially in those patients with varus deviation of the hindfoot.

**Fig. 1:**
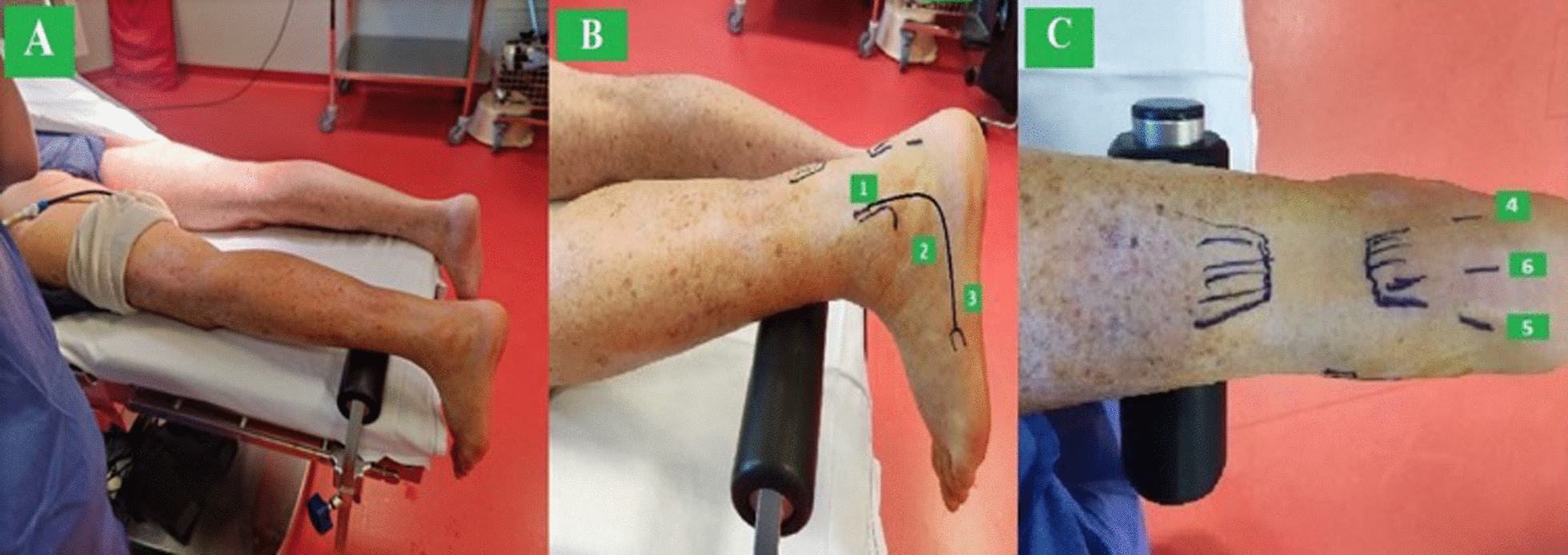
**A** Patient setup in a prone position with ankle support; **B** two endoscopic portals and the working portal (3rd); and **C**: positioning of the 4th, 5th, and 6th portals

With the patient prone under sciatic–femoral nerve block, spinal or general anaesthesia, the feet lie distal to the operating table to allow full dorsiflexion of the ankle to be operated on, which is placed on a support (Fig. 1A).

A 4-mm 30° angle arthroscope is used, and gravity 0.9% NaCl irrigation is provided. Additional equipment includes a 4.5-mm bone/soft tissue shaver, with gravity flow irrigation. An arthroscopic diathermy rod, a mosquito clamp, a small Kocher forceps, and a 4.1-mm Shannon burr, for percutaneous surgery, are also used.

**Fig. 2:**
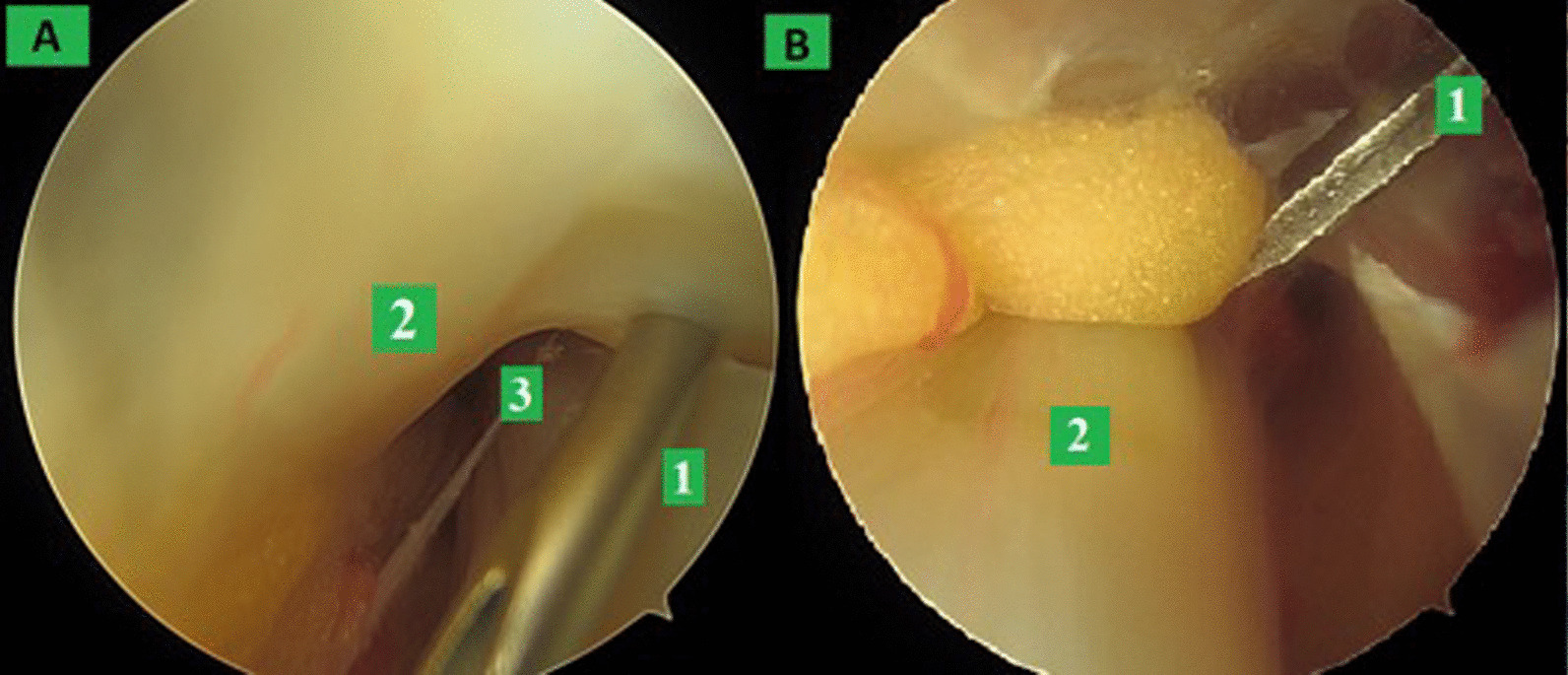
**A** The second portal is positioned at the entrance of the peroneus brevis tunnel with the help of a needle guiding the beaver blade. 1: Needle guiding; 2: tendon pulley; and 3: peroneal brevis tendon (PB); **B** a beaver blade is used to open the pulley of the peroneus brevis. 1: Beaver blade and 2: peroneal brevis tendon (PB)

**Fig. 3:**
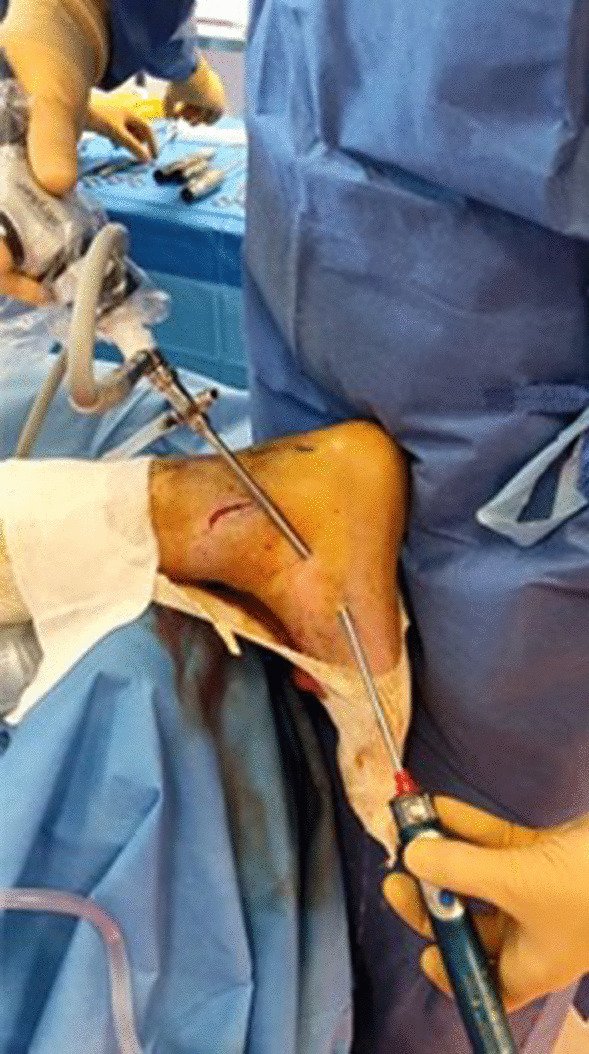
The tendons dissection is performed with a shaver from proximal to distal

The first portal (Fig. 1B) is placed 1-cm proximal and 1-cm posterior to the tip of the fibula. When producing this portal, it is important that, after the skin incision, the subcutaneous soft tissues are released with a trocar or a clip, and the upper peroneal retinaculum is exposed. The 4-mm arthroscope is then introduced, facing distally and anteriorly, thus visualizing the tendons of peroneus brevis and peroneus longus. A septum separates the two tendons, which run into individual tunnels (Fig. 2A). The peroneus brevis tendon is carefully identified as the tendon closer to the surface of the lateral malleolus, and a second portal is produced, with the help of a needle, just at the distal end of the PB tunnel, around 2 cm below the tip of the lateral malleolus. This tunnel is opened using a beaver blade to sever the peroneus brevis pulley (Fig. 2B) at the level of the peroneal tubercle of the calcaneus, and the dissection between the tendons is performed with a shaver from proximal to distal (Fig. 3).

A working portal (Fig. 1B) is produced to release the tendon of the peroneus brevis from its insertion at the base of the 5th metatarsal.

**Fig. 4:**
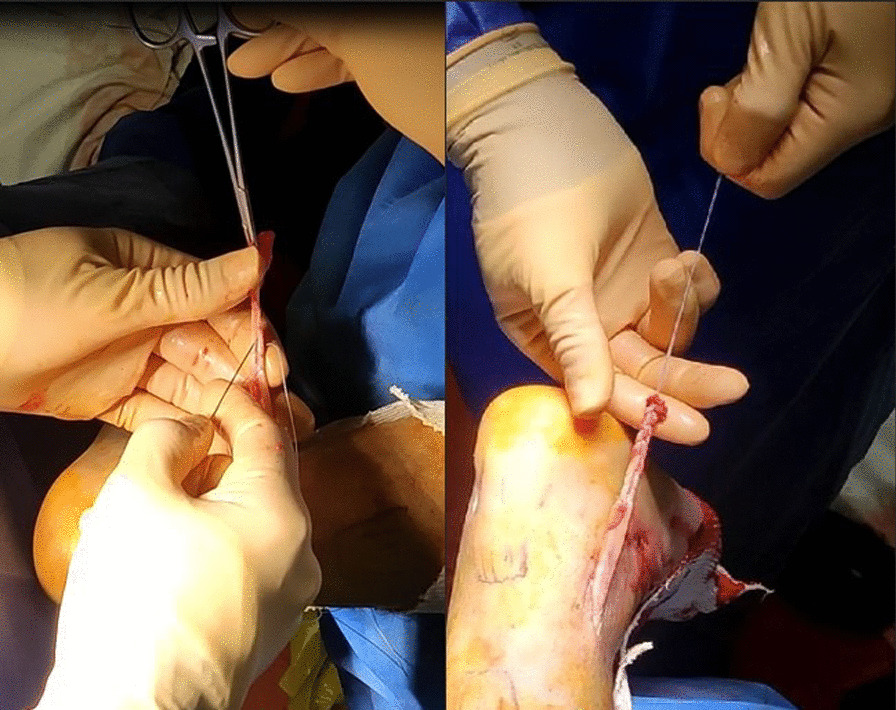
Tendon suture preparation

The tendon is externalized through the first portal, and its free end is whipstitched with FiberLoop (Arthrex) (Fig. 4).

Two portals (Fig. 1C) are produced medial and lateral to the insertion of the Achilles tendon on the calcaneus, and the tendon is freshened using a shaver. The posterolateral calcaneal tuberosity is generously trimmed with a shaver and a Shannon burr so that the transferred tendon will not impinge on the posterosuperior corner of the calcaneum [19, 20]. Also, in this way, the tendon of the peroneus brevis can be transferred more posteriorly, close to the native insertion of the Achilles tendon [10].

**Fig. 5:**
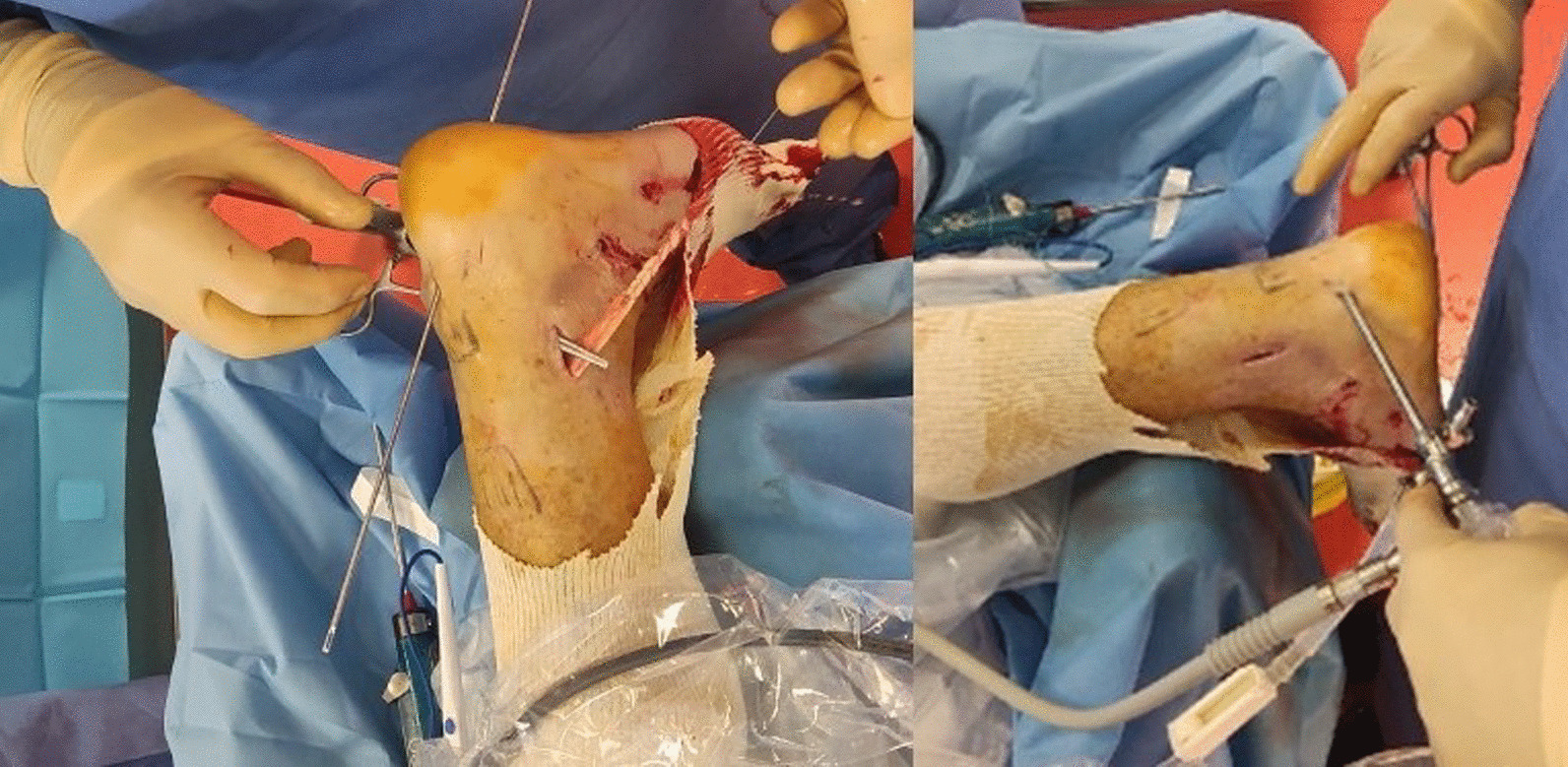
Introduction of the graft into the tunnel and tensioning with Kocher forceps

A sixth portal (Fig. 1 C) is positioned in the middle of the distal calcaneal tendon, and a beath pin is drilled from anterior to the insertion of the Achilles tendon on the calcaneus in a dorsal to plantar direction and from medial to lateral with an angle of 45° through this portal. The pin is over-drilled using a cannulated drill the appropriate diameter according to the size of the patient's tendon (on average, 6 mm). The graft is introduced using a suture relay technique and fixed with an interference screw (7 × 23 mm, Biocomposite, Arthrex) with the ankle in gravity equinus (Fig. 5).

## Postoperative care

**Fig. 6:**
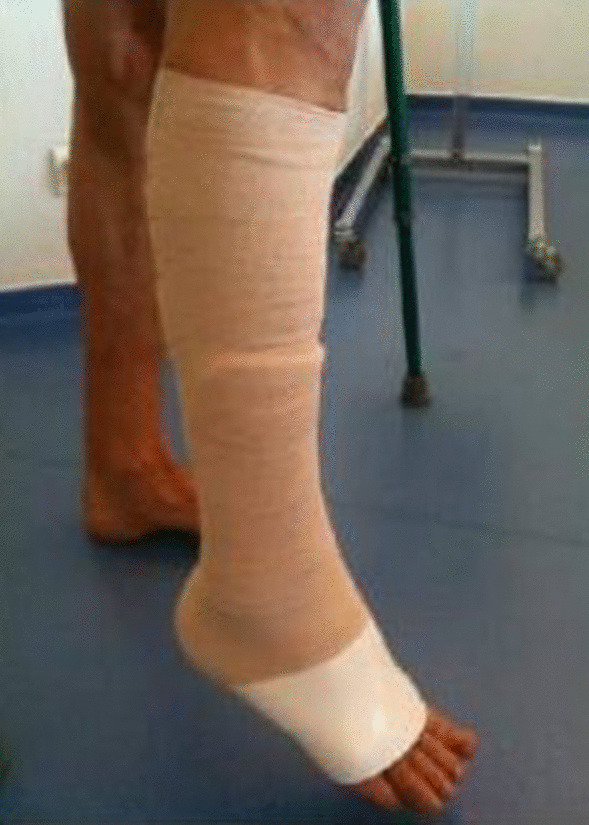
Orthosis in equine position

Thromboprophylaxis is instituted for 6 weeks. Using crutches, the ankle is immobilized in equinus in an anterior splint to prevent dorsiflexion for 3 weeks (Fig. 6), when active and passive dorsiflexion is started [16]. Full range of motion of the ankle and full weight bearing without crutches are started 4 weeks after surgery without orthosis, and full sports activity is started 6 months after surgery. We advise patients of the risk of sural nerve entrapment and neurogenic pain.

## Discussion

Several techniques have been described to manage CATR. Minimally invasive peroneal brevis tendon transfer can be accomplished using a combination of tendoscopy and endoscopy, with a likely lower rate of infection risk using these minimally invasive techniques. Peroneus brevis tendon transfer using mini-open techniques produces good clinical results: the Achilles tendon total rupture score improved significantly from 58 to 91 postoperatively at a mean follow-up of 4.6 years [15].

Transfer of the flexor hallucis longus (FHL) tendon for CATR has been reported with good clinical and functional outcomes, and up to 86% of patients were able to perform a single-heel raise after transfer of the FHL for CATR [17–20]. However, FHL transfer produces a limitation of active range of motion and weakness of peak torque of plantar flexion of the interphalangeal joint of the hallux [21, 22]. The literature reports good results using autologous hamstring tendons as grafts, but head-to-head randomized controlled trials are missing [23]. The peroneus brevis muscle contributes 4% of the total work capacity in plantar flexion and 28% of the eversion capacity of the hindfoot [24]. Once harvested, its action can be at least partially supplemented by the peroneus longus and tertius muscles. The peroneus brevis muscle is an ankle stabilizer, and therefore, this function has to be carefully rehabilitated postoperatively to prevent ankle instability [25]. In fact, the use of the peroneus brevis tendon for the treatment of CART is not recommended in athletes with chronic ankle instability and peroneal tendon injury.

In 2023, Maffulli et al. reported a rate of complications in the use of PB tendon transfer of 7% in 128 patients, five of whom experienced superficial infections and four wound complications [26]. The return to daily activities and the return to sport were only reported in three and four studies, respectively; 79 and 99 patients were evaluated; patients were able to return to daily activities in 13.7 weeks and return to sport in 19.6 weeks. Furthermore, a slower return to sports was reported in patients who underwent PB tendon transfer than those who underwent FHL transfer, but a higher percentage of patients who underwent PB transfer eventually returned to sports than patients who underwent FHL transfer [26].

Further studies are needed to better elucidate the advantages and disadvantages of this technique and to ascertain whether it offers comparable results to the use of free tendon grafts or other local tendon transfers.

## Data Availability

The datasets generated during and/or analysed during the current study are available throughout the manuscript.

## Abbreviations

CATR: Chronic Achilles tendon rupture
PB: Peroneus brevis
FHL: Flexor hallucis longus
